# Mapping the zoonotic niche of Marburg virus disease in Africa

**DOI:** 10.1093/trstmh/trv024

**Published:** 2015-03-27

**Authors:** David M. Pigott, Nick Golding, Adrian Mylne, Zhi Huang, Daniel J. Weiss, Oliver J. Brady, Moritz U. G. Kraemer, Simon I. Hay

**Affiliations:** a Spatial Ecology & Epidemiology Group, Department of Zoology, University of Oxford, Oxford, UK; b Fogarty International Center, National Institutes of Health, Bethesda, Maryland, USA

**Keywords:** Boosted regression trees, Filovirus, Marburg virus disease, *Rousettus aegyptiacus*, Species distribution models, Viral haemorrhagic fever

## Abstract

**Background:**

Marburg virus disease (MVD) describes a viral haemorrhagic fever responsible for a number of outbreaks across eastern and southern Africa. It is a zoonotic disease, with the Egyptian rousette (*Rousettus aegyptiacus*) identified as a reservoir host. Infection is suspected to result from contact between this reservoir and human populations, with occasional secondary human-to-human transmission.

**Methods:**

Index cases of previous human outbreaks were identified and reports of infection in animals recorded. These data were modelled within a species distribution modelling framework in order to generate a probabilistic surface of zoonotic transmission potential of MVD across sub-Saharan Africa.

**Results:**

Areas suitable for zoonotic transmission of MVD are predicted in 27 countries inhabited by 105 million people. Regions are suggested for exploratory surveys to better characterise the geographical distribution of the disease, as well as for directing efforts to communicate the risk of practices enhancing zoonotic contact.

**Conclusions:**

These maps can inform future contingency and preparedness strategies for MVD control, especially where secondary transmission is a risk. Coupling this risk map with patient travel histories could be used to guide the differential diagnosis of highly transmissible pathogens, enabling more rapid response to outbreaks of haemorrhagic fever.

## Introduction

**Figure 1.**
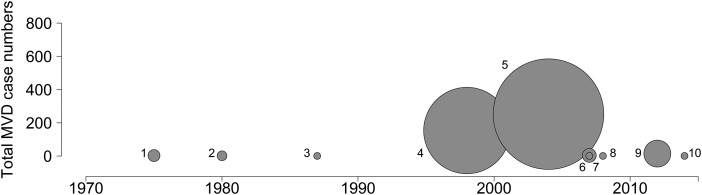
Case numbers in previous Marburg virus disease outbreaks. The size of each circle is proportional to the number of cases of the disease in a given outbreak. Outbreaks are labelled as per Table 1.

In 1967, outbreaks of a previously undescribed disease in workers of three laboratories in West Germany and Yugoslavia were reported, characterised by high fever, haemorrhaging and organ failure.^1^ A novel virus, named Marburg virus (MARV), the first described in the *Filoviridae* family, was subsequently identified as the causative pathogen.^2^ In 1975, the first recognised case of the disease outside of a laboratory occurred in Rhodesia (now Zimbabwe), with one case in 1980 due to MARV and another in 1987 due to Ravn virus (RAVV), another marburgvirus.^3^ Not until 1998, when a series of fatal haemorrhagic cases were identified in the vicinity of Durba, Democratic Republic of the Congo (DRC), was a large-scale outbreak reported. A total of 154 cases were reported, with the source of infection traced back to bat colonies in local gold mines.^4^ While a large number of cases were reported between 1998 and 2000, it was found that multiple introductions of the virus from the same zoonotic pool were responsible for the continued outbreak rather than only human-to-human transmission, as more commonly reported with Ebola virus disease (EVD).^4–6^ In 2004 however, a large outbreak in Uige province, Angola, occurred where, unlike in Durba, continued cases were driven by subsequent human-to-human transmission rather than repeated introductions from a natural source.^7^ More recent outbreaks have been smaller in comparison (Figure 1).^8–12^

**Figure 2.**
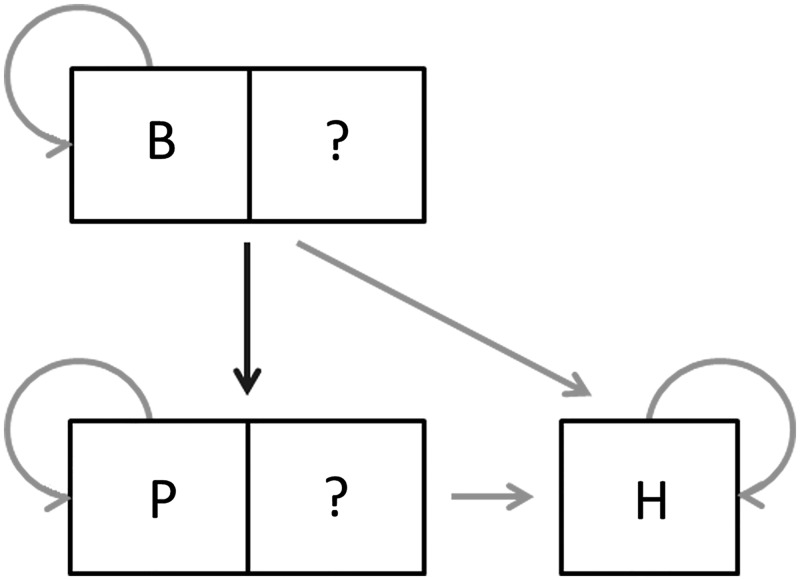
The epidemiology of marburgvirus transmission in Africa. B represents suspected bat reservoirs (including Egyptian rousettes). Susceptible animals include non-human primates, such as the monkeys responsible for the 1967 outbreaks (P). H represents humans. Question marks indicate potential animals of other species. All routes have been confirmed or are suspected to occur apart from transmission between bats and primates, which remains unknown. Adapted from Laminger and Prinz and Groseth et al.^15,16^

The wider epidemiology of Marburg virus disease (MVD) remains relatively unknown (Figure 2). While non-human primates are susceptible to the disease, there have been no reported transmission events from primates to humans outside of a laboratory setting. Furthermore, no significant epizootics have been reported among non-human primates, unlike the closely related ebolaviruses.^13,14^ Past outbreaks have strongly implicated bats as the origin of initial index cases in humans. Serological and molecular surveys conducted in caves and mines visited by the infected individuals, have identified the virus in the Egyptian rousette (*Rousettus aegyptiacus*).^17,18^ Colonies of bats have also been reported in the vicinity of the supposed index case in other outbreaks.^19,20^

In order to better understand the nature of MVD risk, this study attempts to define those areas where zoonotic transmission of MVD may occur in order to identify people at potential risk of zoonotic spillover. Such a methodology has previously been employed with EVD to identify 22 western and equatorial African nations where ebolavirus transmission may occur.^21^ Ecological niche modelling of MVD has previously been undertaken and this work seeks to update these efforts by including more recent outbreaks, records of infection in animals, improved environmental covariate layers and recent advances in modelling techniques.^21–25^ The need for such information is critical, not only to assist in differential diagnosis of fevers across Africa, but also to increase awareness of the potential risk of more widespread outbreaks that could arise from a delay in the response to initial cases.^26^

## Materials and methods

### Methodological overview

A species distribution model, specifically an ensemble boosted regression trees (BRT) framework, was used to model the zoonotic niche of MVD. This model optimally builds ensembles of trees based upon binary decisions used to classify suitable environmental covariates in reference to a database of known occurrence locations.^27,28^ Areas which are environmentally similar to those with reported zoonotic transmission of MVD are predicted to be at higher levels of risk. To perform this analysis, we obtained four key information components: 1. a database of cases where MVD has been transmitted from animals to humans; 2. reported infections of MARV and RAVV in animals; 3. a collection of spatially gridded environmental variables that are likely to be correlates of disease presence; and 4. background (pseudo-absence) data indicating locations where MVD has not been reported. The model was restricted to the African continent since there have been no reported natural outbreaks, in humans or animals, outside this region.

### Identifying human and animal infections with marburgviruses

Outbreaks of MVD in humans were identified from review articles and by sourcing original references.^29^ Where possible, index cases (individuals infected by animal reservoir species) were located and the supposed location of animal to human transfer of MARV and RAVV was geopositioned using Google Earth. When an accurate site location could not be determined, a geographic area (termed a polygon) was defined covering the reported region, identified using the source articles (e.g., a specified landmark, or an area referenced in relation to another directly identifiable site); otherwise a precise, site-specific latitude and longitude was recorded. For larger settlements, the centroid of the site was recorded. In some instances, only the first reported patient could be identified, with little information on the initial route of infection. In these instances we assumed that the index case occurred where the zoonotic transmission event took place. For some outbreaks there was sufficient evidence to suggest multiple independent zoonotic transmission events. For these outbreaks, each individual transmission event was separately positioned.

To obtain a comprehensive database of MARV infections in animals, a literature search was conducted in Web of Science using the search term 'Marburg reservoir OR Marburg monkey OR Marburg bat OR Marburg primate'. This procedure returned 1544 unique citations. Abstracts for these citations were processed and where they indicated that the article might contain spatial information on Marburg infections in animals the full articles were obtained. Once identified, the references of these articles (as well as more general review articles discussing MVD reservoirs) were followed up in case relevant articles were omitted from the initial literature search. Locations of infected animals were geopositioned using the same methodology as for human index cases.

### Covariates used in the analyses

A suite of ecologically relevant gridded environmental covariates for Africa was compiled, each having a nominal resolution of 5 km × 5 km. A number of environmental covariates thought to potentially influence MVD distribution were selected for inclusion in this analysis, namely range and mean values of enhanced vegetation index (EVI) and land surface temperature (LST) (day and night) derived from satellite data and parsed through gap-filling algorithms, as well as elevation and potential evapotranspiration (PET).^21,25,30,31^ Many of these have been considered in previous investigations.^24^ In addition, distance to the nearest Karst formation was included as a covariate.^32^ Karst landscapes typically form when soluble rocks dissolve and can create expansive cave networks and as such were used in the model as a proxy for the subterranean roosting habitat of the supposed disease reservoir, the Egyptian rousette.^33,34^ Previous work mapping the zoonotic niche of EVD utilised a bat distribution covariate layer. While attempts were made to replicate this approach for MVD, the lack of sufficiently detailed data available from the Global Biodiversity Information Facility to allow for differentiation between roosting and foraging sites meant that the niche modelling approaches were unable to produce reliable results and therefore these outputs could not be included in the final analysis.

### Marburg distribution modelling

An ensemble boosted regression trees model was used to define areas environmentally suitable for zoonotic MARV transmission. The model requires both presence and background information to generate a prediction, the latter of which is often hard to collect systematically and in an unbiased manner. As a result, randomly generated background records are often supplied. For this study, a background record dataset was generated by randomly sampling 10 000 locations across Africa, biased towards more populous areas as a proxy for reporting bias.^22^ This sampling allows for comparison of factors influencing presence and likely absence locations for MVD by the model. In total, 500 submodels were used. Each submodel was fitted using the gbm.step subroutine in the dismo package in the R statistical programming environment.^28,35,36^ Given the limited number of records available, we reduced the number of cross-validation folds used to fit the model to three, from the default of 10. All other tuning parameters of the algorithm were held at their default values (tree complexity=4, learning rate=0.005, bag fraction=0.75, step size=10). For each polygon in the occurrence dataset, one point was randomly selected from within the defined area for each submodel. This Monte Carlo procedure enabled the model to efficiently integrate over the environmental uncertainty associated with imprecise geographic data. A bootstrap sample was then taken from each of these datasets and used to train the BRT model. For each submodel, weightings were applied to the background dataset so that the sum of the weighted background data equalled the weighted sum of the occurrence records.^37^ This was done in order to improve the discrimination capacity of the model. Each submodel predicts environmental suitability on a continuous scale from 0 to 1. An ensemble final prediction map was generated by combining the predictions from these submodels, calculating the mean prediction as well as the 5% and 95% confidence intervals around this for each 5 km × 5 km pixel.

Two models were constructed. Model 1 used only records of human index cases and model 2 used both human index cases and reported infection in animals. This was done in order to augment the relatively small number of index case records available and to evaluate the influence of including animal data on the model.

The area under the curve (AUC) statistic was used to assess model accuracy. The statistic was calculated for each submodel using a three-fold cross validation, and then summarised across all the submodels to generate a mean and standard deviation for this value. This procedure divided the dataset into three subsets that had approximately equal numbers of presence records and background data. Due to the small number of presence records used to train each submodel, this approach represents a very thorough test of the model's predictive ability. In order to prevent inflation of the accuracy statistics due to spatial sorting bias, a pairwise distance sampling procedure was used.^38^ As a result, these AUC statistics are lower than typical outputs, but give a more realistic evaluation of the ability of the model to predict for different regions.^39^ Uncertainty in the prediction was evaluated by considering the difference between the 5% and 95% confidence intervals.

The final outputs represent the environmental suitability for zoonotic transmission of MARV for each 5 km × 5 km pixel which allows for relative comparison of risk across Africa.

### Population living in areas of environmental suitability for zoonotic transmission

Estimates of population living in areas at risk of zoonotic transmission were derived by converting the continuous surface of transmission risk into a binary at-risk/not-at-risk classification for each pixel. The threshold for this classification was based upon the minimum environmental suitability value at the locations of the occurrence records. To calculate this value, the risk estimate for each point occurrence and the mean probability of each area/polygon occurrence were assessed. Countries were classified into two categories of risk. Set 1 are countries where index cases of MVD have been reported and set 2 are countries where no index cases have been previously reported and have more than 100 pixels (i.e., approximately 2500 km^2^) at risk. The number of people living in these pixels was calculated from existing population surfaces for Africa.^40,41^

Contiguous areas of risk within each country were visually identified and the latitude and longitude for the approximate mid-point for these areas were recorded, suggesting areas of potential interest for further prospective epidemiological investigation.

The R code used for all of the analysis is freely available via https://github.com/SEEG-Oxford/marburg_zoonotic.

## Results

### Reported infections in humans and animals

**Figure 3.**
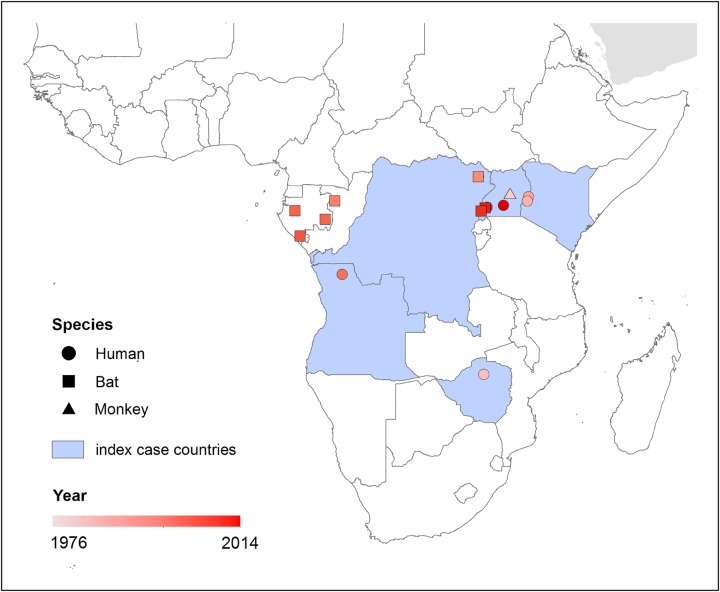
The locations of marburgvirus disease outbreaks in humans and reported animal infections across Africa. This figure is available in black and white in print and in colour at Transactions online.

A total of 10 distinct outbreaks of MVD were identified, ranging in size from single reported cases to community-wide outbreaks with hundreds of reported cases (Table 1). Five countries have confirmed or suspected instances of animal-to-human zoonotic transmission, namely Kenya, Uganda, Zimbabwe, Angola and the DRC (Figure 3). For the majority of these outbreaks, caves or mines have been singled out as the likely venue for their spillover events. Some records were of individuals who had subsequently travelled elsewhere before becoming symptomatic, for which efforts were made to identify the original site of infection.^3,9,10^

**Table 1.** Locations of natural outbreaks of Marburg virus disease in humans

Outbreak	Date range	Countries	Location	Cases/deaths	Reference
1^a^	Feb 1975	Zimbabwe	Chinoyi caves	3/1	3,24
2	Jan 1980	Kenya	Nzoia	2/1	20
3	Aug 1987	Kenya	Kitum cave	1/1	19
4	Oct 1998–Aug 2000	DRC	Durba	154/128	4,42
5	Oct 2004–Jul 2005	Angola	Uige province	252/227	7,43,44,45
6	Jun 2007–Sept 2007	Uganda	Kitaka gold mine	4/1	8
7^a^	Dec 2007–Jan 2008	Uganda	Python cave	1/0	9
8^a^	Jul 2008	Uganda	Python cave	1/1	10
9	Aug 2012–Oct 2012	Uganda	Ibanda district	15/14	11
10	Sep 2014	Uganda	Mpigi/Mawokota district	1/1	12

DRC: Democratic Republic of the Congo.

^a^ Indicates case imported elsewhere.

All available animal infection records were from bat populations, often sampled in response to human cases, with the exception of one reported infection in grivets (the same animals responsible for the 1967 laboratory-based outbreak) (Table 2). These monkeys were trapped near Kidera and Namsale in Uganda where they were assumed to have been originally infected.^46^ Where epidemiological surveys of nearby potential animal reservoirs were undertaken during, or shortly after outbreaks in humans, PCR identification of MARV was often performed.^17,18^ A serological survey of Gabonese bat populations reported positivity in Egyptian rousettes and other bats.^47^ This is the only evidence of animal infection occurring outside the recognised range of reported human populations, all other animal infections have been reported in the vicinity of human outbreaks (Figure 3).

**Table 2.** Locations of reported infections with Marburg virus in animals

Site	Date range	Country	Location	Species	Diagnosis	Reference
1	Aug–Sep 1967	Uganda	Kidera and Namasale	*Chlorocebus aethiops*	Serology	42
2	May–Oct 1999	DRC	Durba	*Miniopterus inflatus*	PCR	16
3	May–Oct 1999	DRC	Durba	*Rhinolophus eloquens*	PCR	16
4	May–Oct 1999	DRC	Durba	*Rousettus aegyptiacus*	PCR	16
5	Jun 2003–May 2006	ROC	Mbomo district	Various bat species	Serology	43
6	Feb 2005–Mar 2008	Gabon	Haut-Ogooue district	Various bat species	Serology	43
7	Apr 2005	Gabon	Moyen-Ogooue district	Various bat species	Serology	43
8	Feb 2006	Gabon	Nyanga district	Various bat species	Serology	43
9	Aug 2007	Uganda	Kitaka gold mine	*Rousettus aegyptiacus*	PCR	15
10	Aug 2007	Uganda	Kitaka gold mine	*Hipposideros* spp.	PCR	15
11	Apr 2008	Uganda	Kitaka gold mine	*Rousettus aegyptiacus*	PCR	15
12	Aug 2009	Uganda	Python cave	*Rousettus aegyptiacus*	PCR	48
13	Nov 2009	Uganda	Python cave	*Rousettus aegyptiacus*	PCR	48
14	Nov 2012	Uganda	Kitaka gold mine	*Rousettus aegyptiacus*	PCR	49

DRC: Democratic Republic of the Congo; ROC: Republic of Congo.

### Predicted environmental suitability for zoonotic transmission of marburgviruses

Due to the relative paucity of data, two model variants were used in order to test various assumptions about the poorly understood MVD epidemiology. Model 1, which only included human index case data, identified geological features (elevation and distance to Karst formation) and vegetation indices (both EVI mean and range) as the main predictors of suitability for zoonotic transmission (Table 3). Model 2, which included the entire dataset of MARV infections, implied a broader spatial extent, with environmental factors (EVI, LST and PET) playing a more important role in prediction compared to elevation. The AUC values were 0.64±0.12 and 0.62±0.08 for models 1 and 2, respectively, indicating that both the models demonstrated similar predictive skill. Note however that as these statistics were calculated using different evaluation datasets, they are not directly comparable. Uncertainty maps for the predicted surfaces for MVD are presented in Supplementary Figures 1 and 2.

**Table 3.** Summary statistics for model outputs

Statistic	Model 1: human data	Model 2: human and animal data
AUC (± standard deviation)	0.64±0.12	0.62±0.08
1^st^ predictor	Elevation: 49.1%	Mean EVI: 49.1%
2^nd^ predictor	Mean EVI: 30.1%	Night-time mean LST: 19.5%
3^rd^ predictor	EVI range: 7.8%	Elevation: 10.9%
4^th^ predictor	Distance to Karst: 4.9%	Mean PET: 7.5%
5^th^ predictor	Night-time mean LST: 3.1%	Day-time mean LST: 5.0%

Relative contributions for each of the top five predictors are reported as a percentage.

AUC: area under the curve; EVI: enhanced vegetation index; LST: land surface temperature; PET: potential evapotranspiration.

Both models predict high suitability for zoonotic transmission in the set 1 countries. In total, model 2 predicts 27 countries to be at-potential-risk (set 1 and 2) of zoonotic transmission of MARV with 105 million people living in at-risk areas. Model 1 predicts 19 countries at risk with 75 million individuals living in at-risk areas. These 19 countries are consistently predicted to be at-risk in both models 1 and 2.

## Discussion

**Figure 4.**
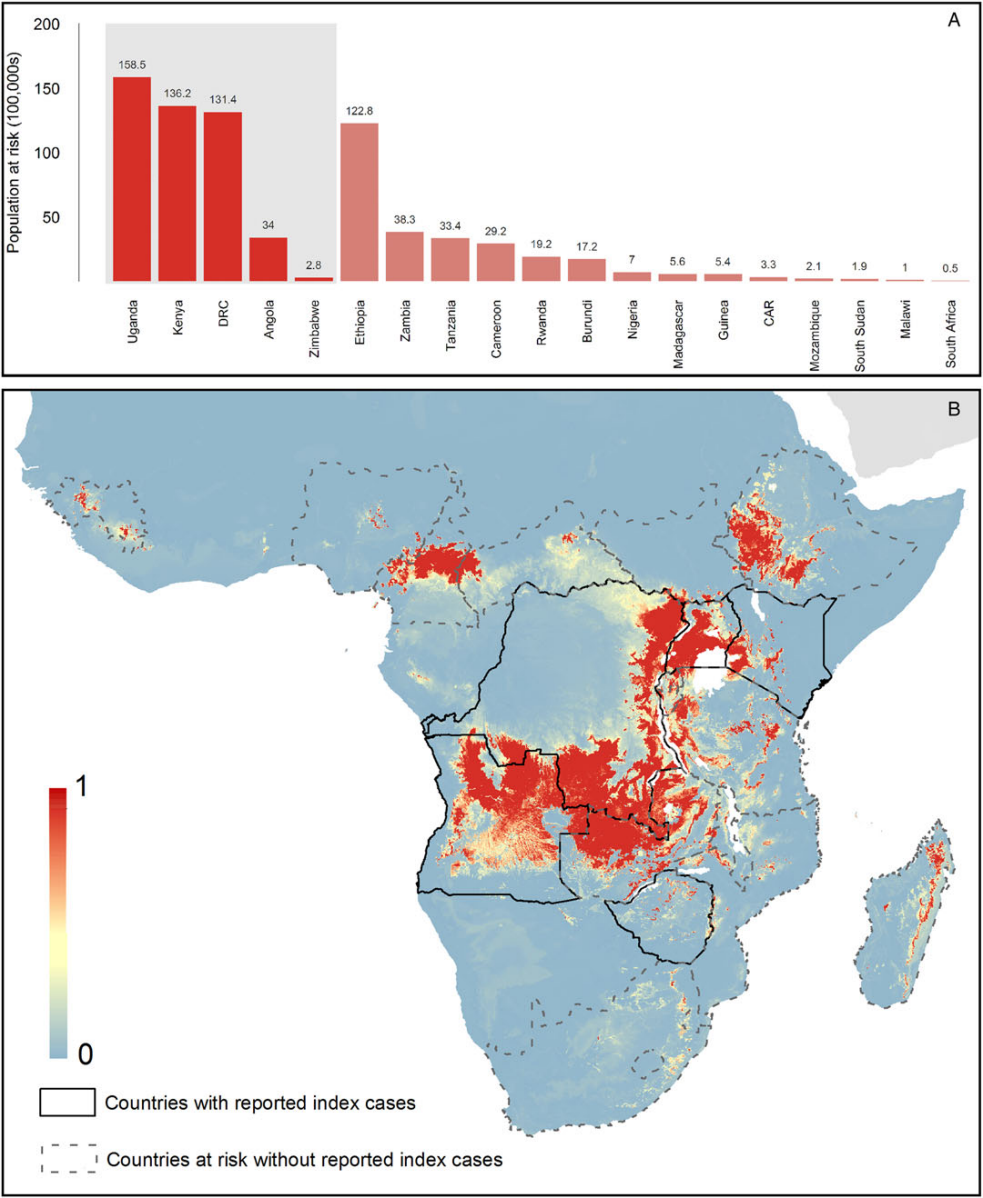
Predicted geographical distribution of the zoonotic niche for marburgviruses using model 1 – human index cases only. Panel A shows the total populations living in areas of risk of zoonotic transmission for each at-risk country. The grey rectangle highlights countries in which index cases of disease have been reported (set 1); the remainder are countries in which risk of zoonotic transmission is predicted, but in which index cases of Marburg virus disease have not been reported and have more than 100 at-risk pixels (set 2). These countries are ranked by population-at-risk within each set. The population-at-risk figure in 100 000 s is given above each bar. Panel B shows the predicted distribution of zoonotic marburgviruses. The scale reflects the relative probability that zoonotic transmission of marburgviruses could occur at these locations; areas closer to 1 (red) are more likely to harbour zoonotic transmission than those closer to 0 (blue). Countries with borders outlined are those which are predicted to contain at-risk areas for zoonotic transmission based on a thresholding approach (see Methods). The area under the curve statistic, calculated under a stringent cross-validation procedure is 0.64±0.12. Solid lines represent set 1 whilst dashed lines delimit set 2. Areas covered by major lakes have been masked white.

**Figure 5.**
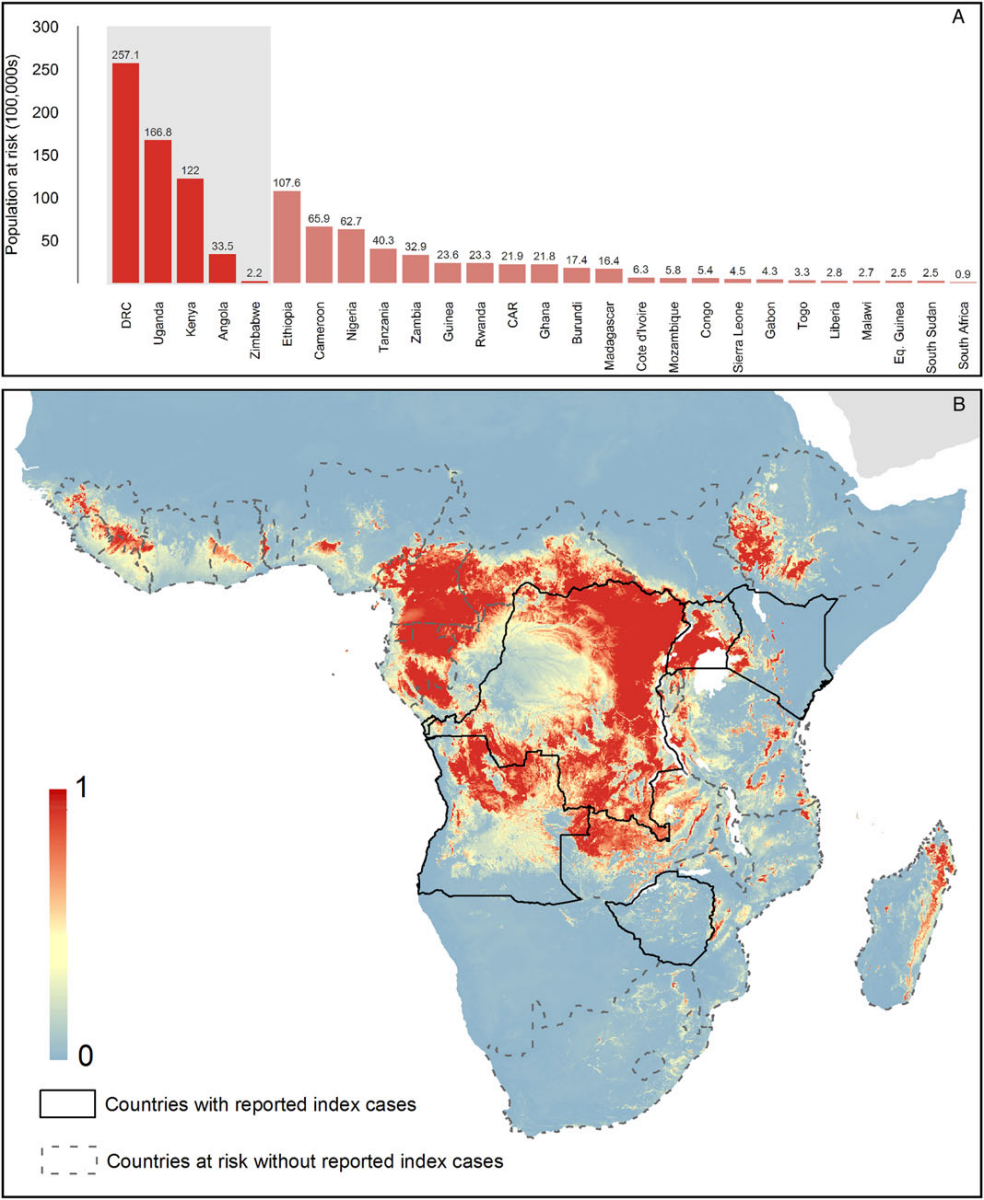
Predicted geographical distribution of the zoonotic niche for marburgviruses using model 2–both human index cases and infections in animals. Panel A shows the total populations living in areas of risk of zoonotic transmission for each at-risk country. The grey rectangle highlights countries in which index cases of Marburg virus disease have been reported (set 1); the remainder are countries in which risk of zoonotic transmission is predicted, but in which index cases of Marburg have not been reported and have more than 100 at-risk pixels (set 2). These countries are ranked by population-at-risk within each set. The population-at-risk figure in 100 000 s is given above each bar. Panel B shows the predicted distribution of zoonotic marburgviruses. The scale reflects the relative probability that zoonotic transmission of marburgviruses could occur at these locations; areas closer to 1 (red) are more likely to harbour zoonotic transmission than those closer to 0 (blue). Countries with borders outlined are those which are predicted to contain at-risk areas for zoonotic transmission based on a thresholding approach (see Methods). The area under the curve statistic, calculated under a stringent cross-validation procedure, is 0.62±0.08. Solid lines represent set 1 whilst dashed lines delimit set 2. Areas covered by major lakes have been masked white.

This work utilises all known outbreaks of MVD in humans and reported infections in animals in order to understand the nature of risk posed by this disease (Figures 4 and 5). Previous assessments have indicated that a much broader region is at-risk of zoonotic transmission than those countries that have reported transmission to-date.^24^ Our analysis, reinforced by new outbreak reports and environmental covariate information, is in concordance with previous ecological modelling investigations of MVD, identifying temperature and vegetation indices as key determinants of its spatial distribution.^23,24,50^ In addition, we identify the potential importance of geological features in influencing areas of potential MARV risk. The majority of at-risk populations live in areas that have previously reported outbreaks, mainly Uganda, Kenya and the DRC. Amongst countries yet to see human infection (set 2), the most notable are Ethiopia, Cameroon and Zambia, in which large areas are predicted to be at-risk.

As with any model-based approach, an awareness of the limitations of the data and the assumptions made by the model is important. Limited datasets, particularly those where definitive identification of zoonotic transmission sites is unlikely, will hinder predictive capability. However, this study attempts to be as comprehensive as possible by including all reports of infections, as well as considering uncertainty in geopositioning ability. Further information can only help to improve these predictions. Similarly, the model is only able to assess environmental suitability for MVD, therefore in order to translate this into true outbreak risk, additional information on how humans and animal reservoirs interact, as well as how the disease is transmitted within these populations is required. Bearing these caveats in mind, we hope that these results will act as a springboard for further research to better understand the epidemiology and characterise the risk of this disease.

**Figure 6.**
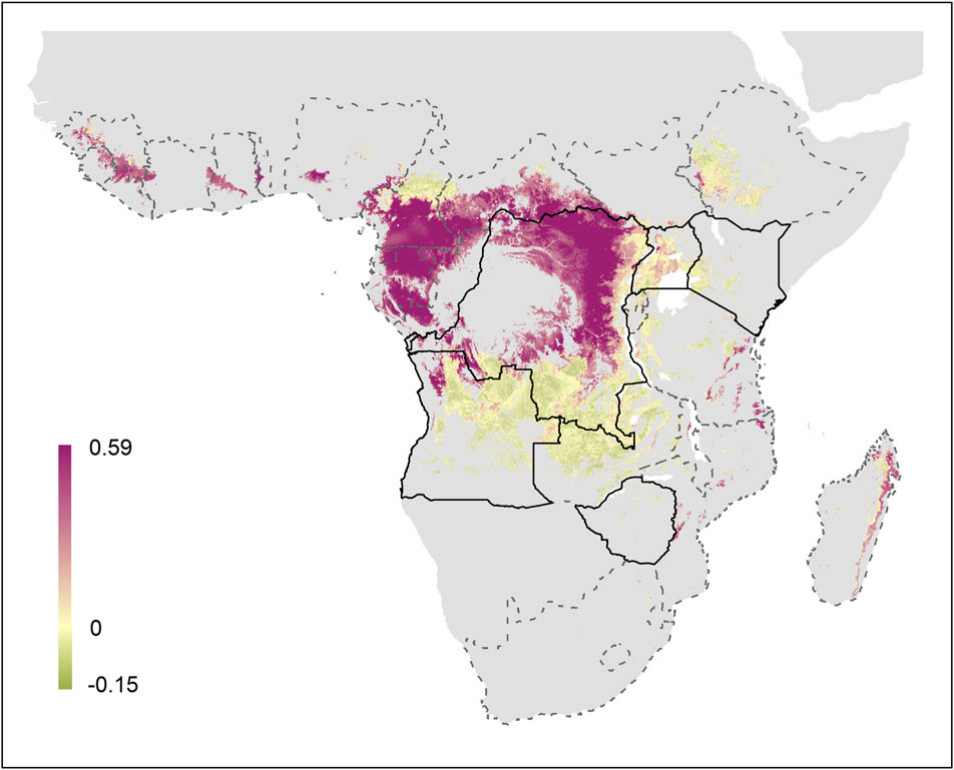
Difference between model predictions with animal data omitted. The difference between outputs for model 2 and model 1 are presented. Pixels in purple represent those regions predicted at higher risk in model 2; regions in green indicate areas where model 1 predicts higher risk. Yellow pixels represent areas with consistent probabilities. Pixels predicted not to be at-risk are in grey.

The two model iterations (‘human only’ versus ‘animal and human data’) illustrate the need for further research into MARV hosts and their potential for zoonotic transmission to humans. Areas predicted at-risk in model 1 are consistently identified at-risk in model 2 (although the absolute probability of transmission is altered); the inclusion of animal data however, expands the areas of potential risk to include countries across western and central Africa not indicated as being at-risk by model 1. Figure 6 visualises the differences between these two models. As a result, given the limited data availability, model 2 would currently be the most sensible option when discussing the potential risk posed by MVD. In addition, while no reported cases of MVD have been recorded in set 2 countries, a number have seen serological evidence of past exposure in humans.^29^ Seropositive individuals have been reported in locations identified as at-risk in model 2 in West Africa, Cameroon, Central African Republic, Nigeria and South Sudan.^48,49,51–56^

Since many MVD spillover events have only resulted in a handful of cases, the likelihood of outbreaks going unrecognised is a possibility.^57^ It is therefore also possible that spatial variation in the probability of cases being identified may have biased our models. While we strived to account for such an observation bias in our analysis by weighting pseudo-absence records to areas where infection might be more likely to be detected, we cannot rule out the presence of residual bias. The true nature of zoonotic transmission potential within these countries can only be elucidated by additional surveys.

Knowledge on the animal reservoir for MVD is limited. Egyptian rousettes have consistently been identified as PCR positive for the virus, however animals of a number of other species have also been seropositive.^15,43,58,59^ The maps presented here can be used to target key sites for future surveys of bats to better understand the true nature of risk within those areas where no previous outbreak has been reported.

**Figure 7.**
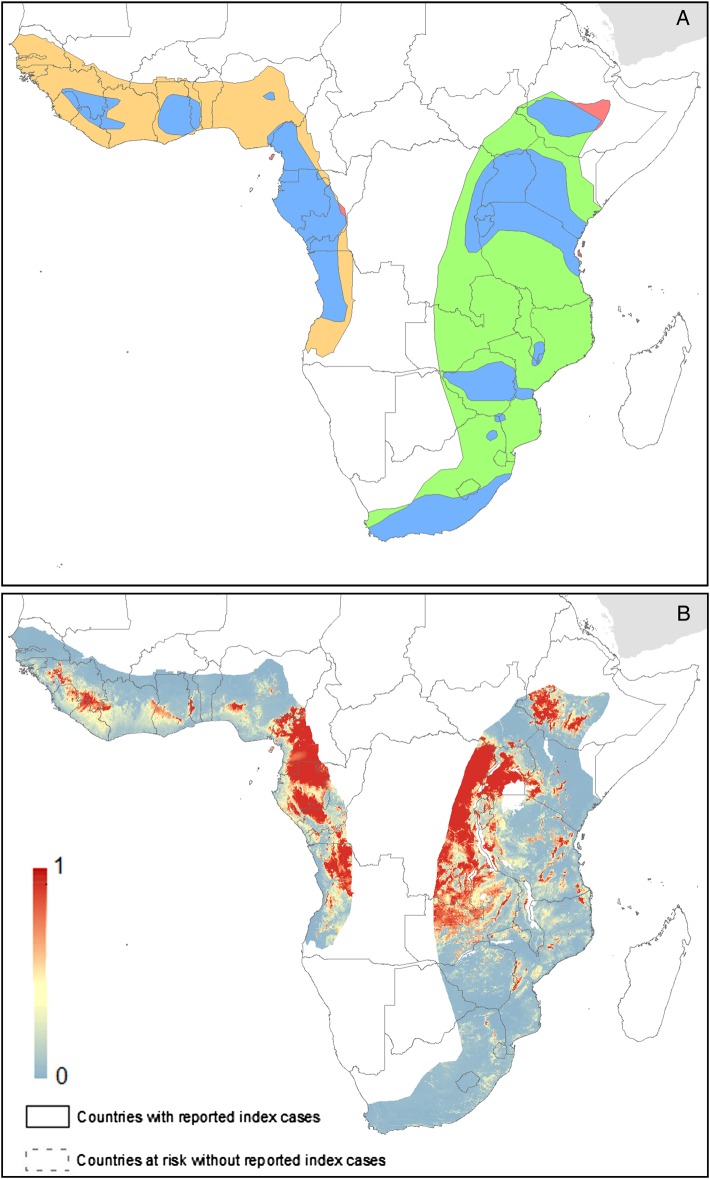
Expert opinion maps for the range of Egyptian rousettes. Panel A is derived from the IUCN and Kwiecinski et al.^33,60^ Blue regions are those where both depict Egyptian rousette populations. Red areas are those only indicated in the IUCN dataset. Orange sections are where bats of the subspecies *R. aegyptiacus unicolor* are thought to be present; green shows the distribution of bats of the subspecies *R. aegyptiacus leachi*. Panel B shows the predicted values from model 2, masked by the bat layer.

There is considerable overlap between the reported distribution of Egyptian rousettes (Figure 7) and areas of highest risk. Evidence suggests that there are various subspecies of Egyptian rousette across Africa.^33^ All but one outbreak of MVD in humans occurred within the known range of members of *R. aegyptiacus leachi*; the outbreak in Uige Province, Angola, however occurred outside the range of bats of this subspecies, but was within the reported range of *R. aegyptiacus unicolor*. It remains unclear whether these populations differ in disease transmission cycles and the nature of the connectivity between bats of these two potential subspecies has important implications for potential disease transmission, either restricting the likely areas of risk to eastern and southern Africa, or including much of central and west Africa (Figure 7B). Similarly, it is possible that bats of subspecies present in north Africa and the Middle East could also be potential reservoirs for MARV. The inclusion of bat distributions in future models would allow for a better understanding of the relationship between MVD and Egyptian rousettes, with the possibility of identifying regions where other bats may be more likely reservoir hosts.

In addition, further surveys for MARV infection in bats in these regions therefore would not only help to better understand the ecology of these bats but also the nature of the risk posed to human populations. As with EVD, spillover of MARV into humans is rare and infection in bat populations also appears uncommon. Understanding the nature of infection within these bat and other potential reservoirs, is crucial in identifying the true nature of risk to human populations, not just for MVD, but also a variety of other viral pathogens.^61^ Table 4 identifies the main regions within each at-risk country where such surveillance activities would be of greatest benefit. The output maps, to allow for national survey placement, are freely available from the following link: http://goo.gl/0qTOfe

**Table 4.** Identification of potential survey sites in at-risk countries

Country	Region	Latitude	Longitude
Angola	Northern Angola	−7.83	16.07
	Moxico	−12.28	23.29
Burundi	Western Burundi	−3.94	29.44
Cameroon	Central and Southern Cameroon	4.73	12.76
CAR	South CAR	5.95	18.91
Côte d'Ivoire	Dix-Huit Montagnes	7.62	−7.89
	Bondoukou	7.96	−3.03
DRC	Eastern Congo Basin	2.04	27.72
	Katanga	−10.70	26.23
	Kasai	−6.42	21.95
	Western DRC	−7.03	18.30
Equatorial Guinea	Kie-Ntem and Wele-Nzas Provinces	1.55	10.83
Ethiopia	Western Oromia	8.29	35.77
	Bale Mountains and Harenna Forest	6.07	39.14
Gabon	Northern Gabon	1.07	12.36
	Southern Gabon	−1.83	11.92
Ghana	Ashanti Uplands and Kwahu Plateau	6.99	−1.61
	Akwapim-Togo Range	8.01	0.51
Guinea	Moyenne Guinea	10.71	−12.47
	Guinea Highlands	8.23	−9.09
Kenya	Lake Victoria	−0.70	34.99
	Mount Kenya	−0.43	37.49
Liberia	Guinea Highlands–Wologizi and Wonegizi Ranges	8.15	−9.87
	Guinea Highlands–Nimba Range	7.41	−8.59
Madagascar	Tsaratanana and Marojejy Nature Reserves	−14.21	49.38
	Ambohijanahay Reserve	−18.38	45.45
Malawi	Lake Malawi	−11.89	33.90
Mozambique	Maeda Plateau	−11.44	39.63
	Mount Mabu	−16.30	36.54
	Inyanga Mountains	−19.64	33.29
Nigeria	Ekiti	7.76	5.09
	Gashaka Gumti National Park	7.36	11.57
ROC	Niari and Lekoumou	−2.92	13.19
	Sangha	1.56	14.53
	Likouala	3.17	16.92
Rwanda	Eastern Rwanda	−2.14	30.37
Sierra Leone	Loma Mountains	8.99	−10.98
South Africa	Woodbush Forest Reserve and Motlatse Canyon	−23.94	30.12
South Sudan	Imatong Mountains	3.95	32.64
	Yambio	4.57	28.29
Tanzania	Tanga	−5.94	37.57
	Mahale Mountain National Park	−6.49	30.30
	Kigoma	−4.49	30.64
	Morogoro	−9.58	35.60
Togo	Akwapim-Togo Range	7.59	0.79
Uganda	Great Lakes Region	0.18	31.01
Zambia	Northwestern Zambia	−12.80	24.76
	Northern Zambia	−9.49	29.23
	Muchinga Escarpment	−11.89	31.84
Zimbabwe	Chinoyi	−17.36	30.13

Sites are identified by country. The latitude and longitude represent the centroid of the proposed survey area.

CAR: Central African Republic; DRC: Democratic Republic of Congo; ROC: Republic of Congo.

Our risk maps provide a baseline estimate for the extent of the zoonotic niche of MVD, which can subsequently be enhanced through more specific research. While an area may have the potential for zoonotic transmission of MVD, if humans rarely interact with these animal hosts, spillover events are unlikely to occur. As a result, in spite of a large number of individuals living in areas where transmission is possible, a considerably smaller number will be at-risk of encountering an infected reservoir and subsequently being infected. Surveys and ethnographic assessments can help better understand the true nature of risk within these regions, particularly important if a quantitative assessment of outbreak likelihood is wanted. While environmental triggers have been linked to outbreaks of MVD, equally important in determining outbreak potential is an understanding of the dynamics of the virus within reservoir populations, which has also been shown to be highly variable.^44,58,62^ In attempting to predict outbreaks it is therefore crucial to understand the interplay between environmental factors, human pressures and reservoir host dynamics.^63^

The differences in areas predicted to be at-risk of infection by models 1 and 2 may in fact reflect the manner in which humans interact with bats. Since the majority of human outbreaks have arisen from contact in caves or other underground systems (rather than in the forest foraging sites of the bats, where animal infections have been reported), the risk map derived from model 1 could be a spatial representation of this transmission risk, as opposed to reflecting the broader distribution of infection in animal populations. Further infection surveys and ethnographic research can help to elucidate and map the risk of animal-human transmission within the at-risk region we have identified. Such an analysis would be particularly important in order to produce an absolute, rather than relative estimate of the likelihood of an outbreak in humans.

Nevertheless, while the true nature of risk to humans is likely to be a function of a variety of different factors, it is still important to gauge how and where potential spillover events could occur. The west African outbreak of EVD has shown that it is critical to understand the potential for such outbreaks in geographically distinct areas, and the subsequent need for other causes to be included in the differential diagnosis to facilitate rapid detection. This is all the more important where the potential causes of disease have varying potential for nosocomial transmission, as is the case with viral haemorrhagic fevers. Failure to rapidly and accurately diagnose these diseases can lead to uncontrolled chains of secondary transmission in certain scenarios.^64^ Maps such as ours can therefore be used to shape clinical recommendations for diagnosing haemorrhagic fever cases presenting in hospital. MVD has seen a number of significant geographic translocation of cases, with individuals becoming symptomatic far from the original infection site.^9,10^ The most recent outbreak of EVD in west Africa has demonstrated the role that both local and global connectivity can play in causing disease importation, and as connectivity continues to increase, the likelihood of widespread secondary cases occurring will also increase, particularly if infection reaches densely populated areas.^65–67^ Accounting for a range of possible aetiological agents can therefore reduce the risk of further secondary transmission amongst humans in these settings.^3,26^

## Supplementary data

Supplementary data are available at Transactions Online (http://trstmh.oxfordjournals.org).

Supplementary Data
