# Supplementary material for: Mapping the zoonotic niche of Marburg virus disease in Africa
Source: Trans R Soc Trop Med Hyg. 2015 Mar 27;109(6):366–78. doi: 10.1093/trstmh/trv024 (PMC4447827; doi:10.1093/trstmh/trv024)
Supplement: Supplementary Data [file supp_109_6_366__index.html]

Mapping the zoonotic niche of Marburg virus disease in Africa — Supplementary Data 

# Mapping the zoonotic niche of Marburg virus disease in Africa

## Supplementary Data

Supplementary Data

**Files in this Data Supplement:**

- Supplementary Data - Docx file
