# Supplementary material for: Mapping the zoonotic niche of Marburg virus disease in Africa
Source: Trans R Soc Trop Med Hyg. 2015 Mar 27;109(6):366–78. doi: 10.1093/trstmh/trv024 (PMC4447827; doi:10.1093/trstmh/trv024)
Supplement: Supplementary Data [file supp_trv024_trv024supp.docx]

**
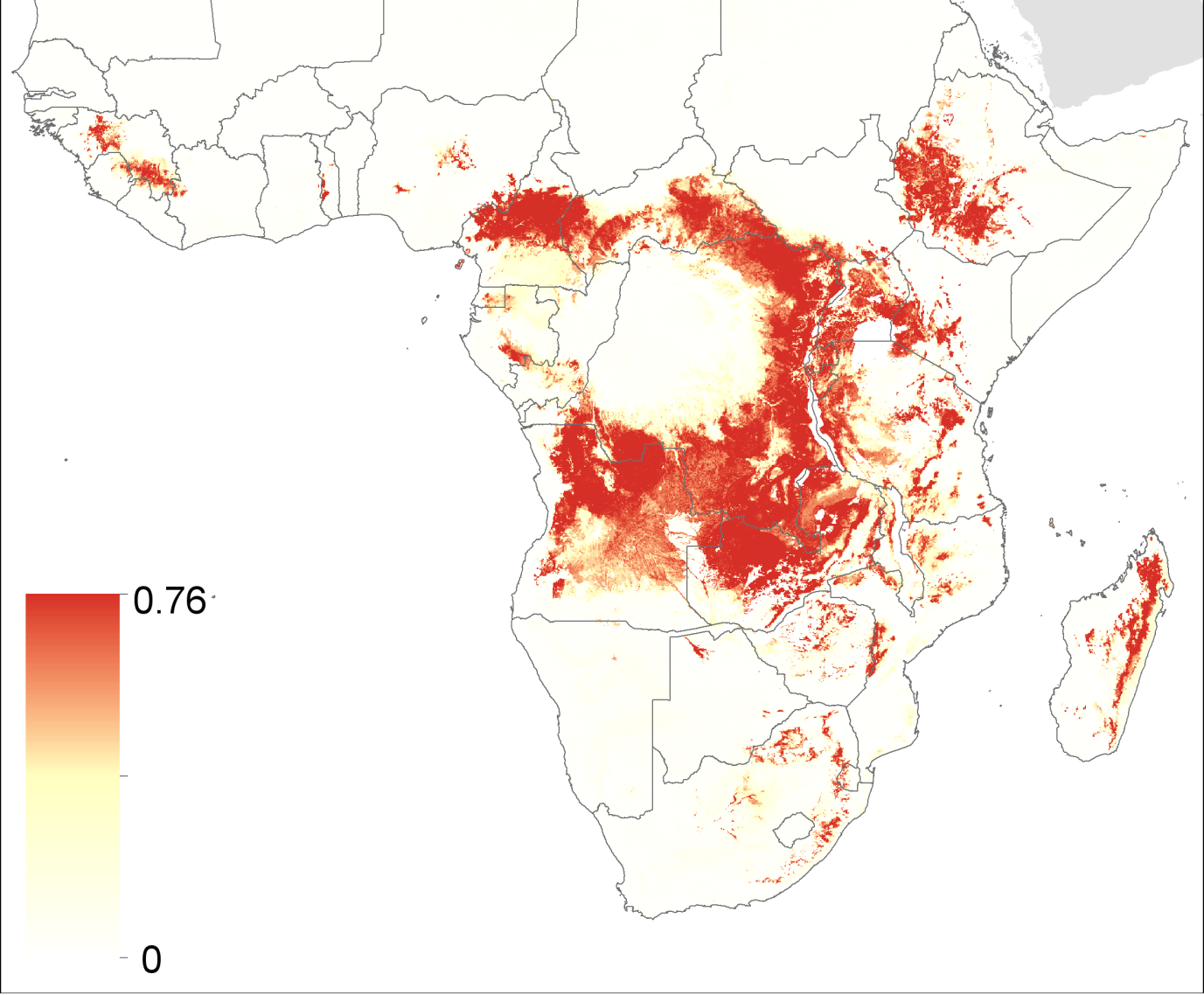
**

**Supplementary Figure 1. Prediction range of model 1: human cases only.** The difference between the 5 and 95% confidence interval of predicted values was calculated. Areas in red have the greatest range in prediction values whilst areas in white, the smallest. The maximum range of pixel values is 0.76.

**
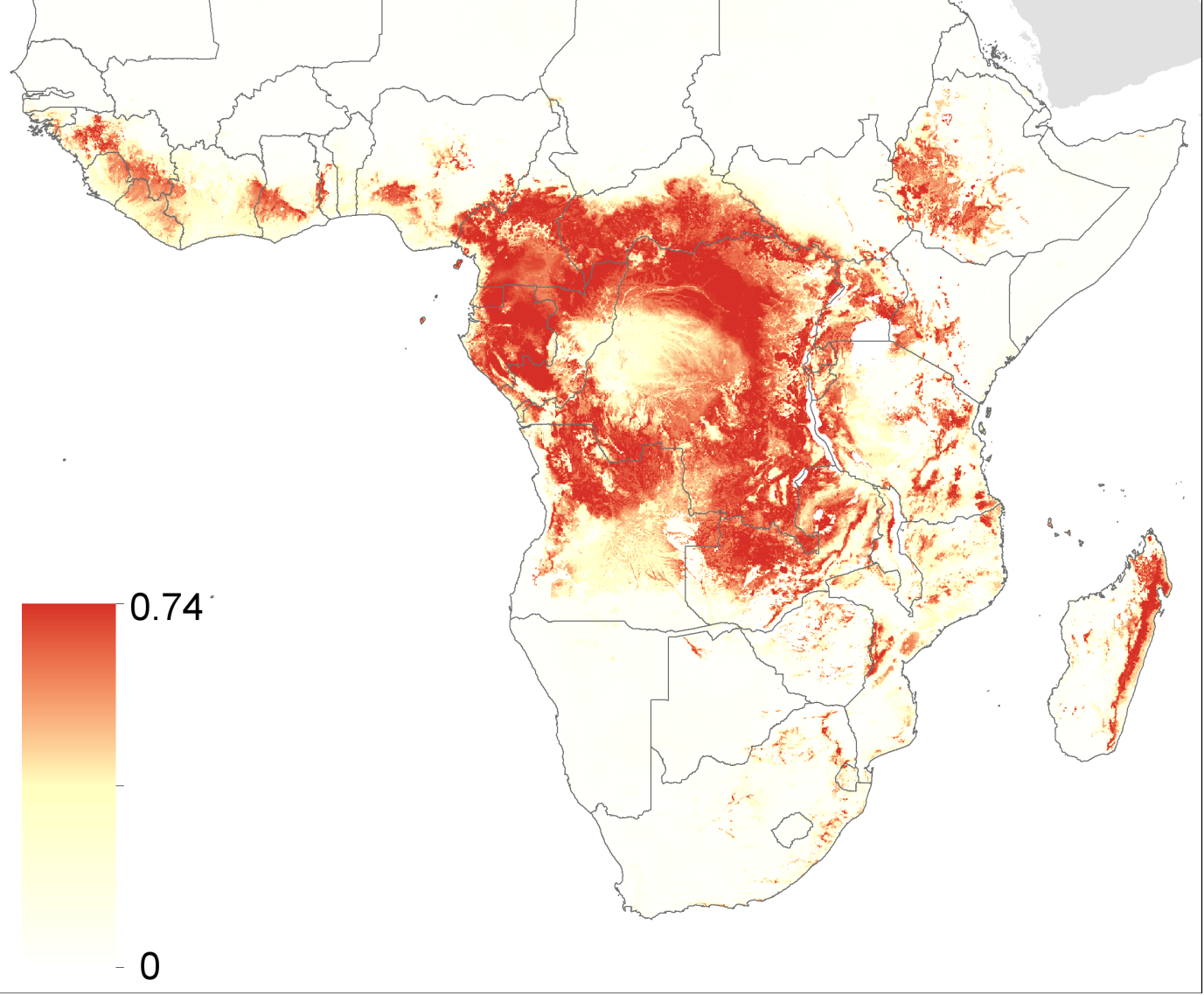
**

**Supplementary Figure 2. Prediction range of model 2: both human index cases and infections in animals**. The difference between the 5 and 95% confidence interval of predicted values was calculated. Areas in red have the greatest range in prediction values whilst areas in white, the smallest. The maximum range of pixel values is 0.74.
